# Central aspects when implementing an electronic monitoring system for assessing hand hygiene in clinical settings: A grounded theory study

**DOI:** 10.1177/17571774241230678

**Published:** 2024-02-05

**Authors:** Karin Granqvist, Linda Ahlstrom, Jon Karlsson, Birgitta Lytsy, Annette Erichsen

**Affiliations:** 1 Department of Anaesthesia, Surgery and Intensive Care, Sahlgrenska University Hospital, Gothenburg, Sweden; 2 The Sahlgrenska Academy, Institute of Health and Care Sciences, University of Gothenburg, Gothenburg, Sweden; 3 Department of Orthopaedics, Sahlgrenska University Hospital, Region Västra Götaland, Gothenburg, Sweden; 4 Department of Orthopaedics, Institute of Clinical Sciences, Sahlgrenska Academy, University of Gothenburg, Gothenburg, Sweden; 5 Department of Laboratory Medicine, Division of Clinical Microbiology, Karolinska Institutet, Sweden

**Keywords:** Hand hygiene, guideline adherence, monitoring technology, grounded theory, implementation science, human technology interaction

## Abstract

**Background:**

New technologies, such as electronic monitoring systems, have been developed to promote increased adherence to hand hygiene among healthcare workers. However, challenges when implementing these technologies in clinical settings have been identified.

**Aim:**

The aim of this study was to explore healthcare workers’ experiences when implementing an electronic monitoring system to assess hand hygiene in a clinical setting.

**Method:**

Interviews with healthcare workers (registered nurses, nurse assistants and leaders) involved in the implementation process of an electronic monitoring system (*n* = 17) were conducted and data were analyzed according to the grounded theory methodology formulated by Strauss and Corbin.

**Results:**

Healthcare workers’ experiences were expressed in terms of *leading and facilitating, participating and contributing,* and *knowing and confirming*. These three aspects were merged together to form the core category of *collaborating for progress*. Leaders were positive and committed to the implementation of the electronic monitoring system, endeavouring to enable facilitation and support for their co-workers (registered nurses and nurse assistants). At the same time, co-workers were positive about the support they received and contributed by raising questions and demands for the product to be used in clinical settings. Moreover, leaders and co-workers were aware of the objective of implementing the electronic monitoring system.

**Conclusion:**

We identified dynamic collective work between leaders and co-workers during the implementation of the electronic monitoring system. Leadership, participation and knowledge were central aspects of enhancing a collaborative process. We strongly recommend involving both ward leaders and users of new technologies to promote successful implementation.

## Background

Hand hygiene (HH) is regarded as an effective measure to prevent healthcare-associated infections (HAI) and is thus one of the most crucial safeguards for patient safety (Allegranzi and Pittet, 2009; Pittet et al., 2000). Despite this, adherence to HH among healthcare workers (HCWs) has been suboptimal for many years (Erasmus et al., 2010; Lambe et al., 2019). Direct observations to monitor HH events, conducted by trained observers, are used in healthcare settings to promote increased adherence (World Health Organization, 2009), but they have been criticized for being time consuming, requiring substantial resources and capturing only a small amount of the total HH events performed (Boyce, 2017; Jeanes et al., 2019). Also, the fact that an observer will encourage those observed to perform even better, the so-called Hawthorne effect, cannot be ignored (Hagel et al., 2015). To promote objective ways of assessing HH and less resource-intensive methods, new technologies, such as electronic monitoring systems (EMS), have been developed in recent years (Boyce, 2017; Marra and Edmond, 2012; Ward et al., 2014). However, implementing technologies for assessing HH adherence in healthcare settings have revealed challenges (Conway, 2016; Ward et al., 2014). Choosing a technical system that best fits the organization and its workflow has been identified as challenging, as well as getting the HCWs to trust these new technical systems (Conway, 2016; Ward et al., 2014).

There is consistent evidence indicating that infection prevention interventions, when combined with a well-planned implementation strategy, have proven successful in reducing HAIs within clinical settings (Price et al., 2023). This success can be attributed to the fact that implementation strategies not only encompass the technical aspects of the intervention but also consider how to effectively manage the social and contextual factors that influence the implementation process (Gilmartin and Hessels, 2019).

In the realm of implementation science, researchers have placed considerable emphasis on identifying various factors that contribute to the success or failure of an implementation process. These investigations have recognized participants' experiences as crucial components in understanding why and how implementation efforts can either succeed or fail (Nilsen, 2015). Nevertheless, few studies have focused on HCWs’ experiences when implementing an EMS in clinical settings (Conway et al., 2014; Kelly et al., 2021; Tarantini et al., 2019). The aim of this study was to explore HCWs’ experiences when implementing an EMS to assess HH in a clinical setting.

## Method

### Design

This study is part of an external evaluation of the implementation of an EMS. The entire implementation was initiated and performed by the product developer and HCWs on the surgical ward, where the EMS was installed, without involvement from the researchers.

The present qualitative study is based on grounded theory (GT), as described by Strauss and Corbin (Strauss and Corbin, 2015) and follows guidelines for qualitative research (Standards for Reporting Qualitative Research, SRQR) (O'Brien et al., 2014), see supplementary file.

### The electronic monitoring system

The EMS, Tork Hand Hygiene Compliance Monitoring System (Essity AB, Sweden), is a position system designed to automatically assess HH adherence. The system consists of individual tags worn by the users, antennae installed in the ward ceiling and internet-connected automatic hand disinfection dispensers. The individual HCW wears a tag during work sessions, which registers, in real time, movements in and out of virtual zones created on the ward at different positions, in each room or around each patient bed. The zones are based on where patient care is provided on the ward. The tag also documents the use of hand disinfection from internet-connected dispensers, before and after entering a virtual zone. This enables the EMS to determine whether or not the participants have used hand disinfection before and/or after entering a zone. Data from the system are stored in cloud-based information technology, and digital feedback to HCWs consists of HH adherence rates provided by the system. The feedback can be given either at group level (via a screen in the nurses’ office, in real time) or at individual level (optionally via personal e-mail or text messages, delivered every week).

### Context and implementation of the electronic monitoring system

The EMS was installed in different phases on a surgical ward at a university hospital in Sweden in October 2018–March 2020 (Table 1). Participating and using the EMS was voluntary.

**Table 1.** The electronic monitoring system was installed on a 24-h open surgical ward (22 beds) at a large university hospital in Sweden. The entire implementation process was initiated and organized jointly by the surgical ward and the product developer and was carried out in different phases.

Phase 1	Phase 2	Phase 3	Phase 4
May 2017–Sept 2018	Oct 2018–Dec 2018	Dec 2018–Feb 2019	Feb 2019–March 2020
EMS installed in four patient rooms on the ward (pilot implementation project)	EMS installed on the entire ward	Feedback provided at group level	Feedback provided at individual and group level
2–5 users	20–25 users	20–25 users	20–25 users

EMS, electronic monitoring system.

### Participants and data collection

The HCWs on the ward were registered nurses (RNs) and nurse assistants (NAs), including one ward manager (RN). Two of the NAs served as facilitators and had been involved when implementing the EMS. They were tasked with running the project at ward/user level. As in GT, theoretical sampling was used in the selection of participants (Strauss and Corbin, 2015). A total of 23 HCWs used the EMS and wore a tag (December 2019) and, of these, 17 HCWs were interviewed. A total of 15 participants had used the EMS from the beginning of implementation on the entire ward (October 2018), while two of them had been users for less than 1 year. Among the participants, one ward manager and two facilitators of the project were included. Hereinafter, we define ward manager and facilitators as leaders at ward level and the remaining participants as co-workers.

Interviews were conducted between December 2019 and November 2020 by the first author (KG) and were based on participants’ choice as individual, pair or group interviews. All the interviews took place at the participants’ convenience and were recorded by the interviewer. Two individual interviews, one pair interview and one group interview (three participants) were conducted face to face. The remaining interviews (*n* = 10) were then conducted as individual interviews via telephone. The interviews were transcribed verbatim. Transcripts and recordings were scrutinized and discussed within the research team (KG, LA, AE). Concepts were deemed to be saturated when 17 HCWs (9 RNs and 8 NAs) had been interviewed.

### Ethical considerations

The participants were informed both in writing and orally about the aim and design of the study and that participation was voluntary and confidential. All the participants provided informed consent before the interviews were conducted. The study was approved by the Swedish Ethical Review Authority (Dnr: 2019-02574).

### Data analysis

The analysis was performed as an iterative process via constant comparison of the data and was carried out in three steps: initial coding, axial coding and selective coding (Strauss and Corbin, 2015). A program for qualitative data (NVivo 12 QSR International) was used as an administrative support in the analysis work. For a detailed description of the data analysis, see the supplementary file.

## Results

**Figure 1.**
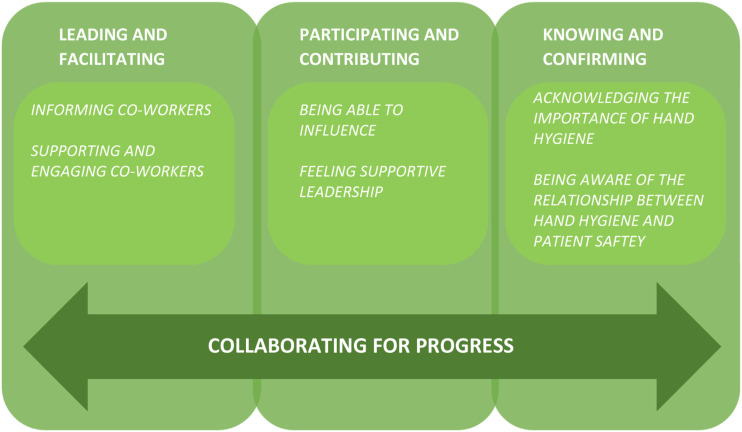
Collective actions taken by leaders and co-workers to enhance a collaborative progress when implementing an electronic monitoring system in a clinical setting.

The analysis resulted in three categories: *leading and facilitating, participating and contributing* and *knowing and confirming*. The categories were joined together to identify the core category, *collaborating for progress*, which explains the HCWs’ key approach in the process of implementing a new technology for assessing HH adherence in clinical settings (Figure 1). The categories are presented and enriched with quotations from the HCWs to clarify the meaning of each category (Table 2).

**Table 2.** Quotations derived from the interviews with the healthcare workers, to clarify the categories.

Quotation number	Category source	Sub-category source	Quotation translated to English
Q1	Leading and facilitating	Informing co-workers	‘Information was essential. You think you are giving enough information, but, when you ask, it is clear that they would have liked more information in any case. You need to be aware of this and, once they were given a little more information, I think it worked really well’.
Q2	Leading and facilitating	Informing co-workers	‘I think I have probably talked a great deal about ‘we’. I see it as much more a question of we. I say, for example, that “**we** need to improve.” I don’t say “**you** need to improve.”’
Q3	Leading and facilitating	Informing co-workers	‘We discussed this at a workplace meeting on the ward and we tried to talk about it (possible skin reactions). We didn’t hide anything… we dealt with every single issue. So there were no questions we felt were ignored. We took real responsibility for checking facts and giving feedback. So that they (co-workers) actually felt that “We can raise questions and they (the leaders) will deal with them.”’
Q4	Leading and facilitating	Supporting and engaging co-workers	‘… it’s really good to have a few (facilitators) who are out working with the patients, who are involved during the day, night and weekends. They are the ones who can make an enormous difference’.
Q5	Leading and facilitating	Supporting and engaging co-workers	‘Absolutely, but we have supported the entire management of the project’.
Q6	Leading and facilitating	Supporting and engaging co-workers	‘But I also think about the fact that the company has been encouraging… I think that they have been visible on the ward and this has been a major advantage’.
Q7	Participating and contributing	Being able to influence	‘I think the things we have mentioned have improved’.
Q8	Participating and contributing	Being able to influence	‘To begin with, it was “hang on, what’s this?” and “what do they actually want?”, as we have never had any project like this before, especially when it comes to a company. But when that disappears and you realize that it isn’t a question of selling something, but that we have all had the opportunity to influence things, I think things changed. I suppose it would be true to say that there was tremendous interest’.
Q9	Participating and contributing	Feeling supportive leadership	‘They (the leaders) were also easy to talk to and they explained things in the right way. So it worked. They were also so positive’.
Q10	Participating and contributing	Feeling supportive leadership	‘Well, it would be true to say that, when they (the leaders) were on the ward, they were so enthusiastic, so that… and they are extremely energetic people’.
Q11	Participating and contributing	Feeling supportive leadership	‘People haven’t talked about it (the EMS) quite as much as they did before. In fact, it was previously brought up at every morning meeting’.
Q12	Knowing and confirming	Acknowledging the importance of hand hygiene	‘Yes, to reduce the spread of infection, for example. That’s what I think about, ensuring good hand hygiene to reduce the spread of infection’.
Q13	Knowing and confirming	Acknowledging the importance of hand hygiene	‘But I think we talked much more actively about the basic hygiene routines as a whole’.
Q14	Knowing and confirming	Being aware of the relationship between hand hygiene and patient safety	‘Well, it’s a question of not exposing patients to unnecessary risks, regardless of what it involves – the risk of infection or the risk of falling, for example’.

### Leading and facilitating

Two sub-categories from the perspective of HCWs representing leaders were identified during the analysis:

• Informing co-workers
• Supporting and engaging co-workers

#### Informing co-workers

Regular information to co-workers, both in writing (weekly) and verbally (briefly on weekdays), was described as significant and important when it came to inviting co-workers to participate in the implementation process. Despite this, leaders nevertheless felt that there was a need for even more information to co-workers (Table 2: Q1).

The information consisted of news about the implementation project or practical use of the EMS, as well as reviews of the results from the group feedback. The results were used to encourage, clarify and discuss adherence rates among the co-workers. Leaders felt it was of great importance to improve the close collaboration between the team, as well as within the team group (Table 2: Q2).

Being able to respond immediately to queries, questions and concerns was central to involving all the co-workers. Leaders spoke of not ignoring the co-workers’ doubts or questions. For example, some of the co-workers noticed possible skin reactions on their hands due to the disinfectant from the dispensers. This disinfectant was new and for use only in the monitoring system dispensers and it therefore replaced the disinfectant previously used on the ward. The co-workers’ concerns were taken seriously by the leaders and, in response to a request from the co-workers, the previous disinfectant was reintroduced. This was considered crucial by the leaders in order to make the co-workers continue using the system and its dispensers (Table 2: Q3).

#### Supporting and engaging co-workers

Leaders talked about ‘doing this together’ and expressed the need for support from each other while initiating and implementing the EMS. They also spoke of the importance of being engaged and committed. In turn, this engagement led to a positive feeling of being supported by one another.

The importance of having facilitators on the ward was discussed in detail, in order to work closely with other co-workers and to be able to push the project forward (Table 2: Q4).

On the other hand, the support higher up the leadership hierarchy was not considered to be as essential as the support at ward level. The support at ward level was considered to be completely sufficient. At the same time, ward leaders were well aware of the support they had given during the implementation (Table 2: Q5).

Ongoing support from the company supplying the EMS was described as important and positive (Table 2: Q6). During the implementation, the company made several visits to the ward and was available for questions and queries. This was seen as encouraging by both leaders and co-workers.

### Participating and contributing

From the perspectives of the HCWs using the EMS and being part of the implementation (co-workers), the following sub-categories emerged:

• Being able to influence
• Feeling supportive leadership

#### Being able to influence

Co-workers appreciated their opportunity to influence the implementation process. For example, the replacement of disinfectant and the need for regular information and feedback were mentioned as positive factors. They spoke of being listened to and that it was possible to express one’s individual opinion. Changes were made according to requests from the co-workers (Table 2: Q7).

Some co-workers were skeptical at the start of the project, questioning the use and function of the EMS. This changed to a more positive opinion over time, mainly because of experience of having an impact on the progress of the project (Table 2: Q8).

Co-workers were also positive about the frequent visits made by the company to the ward. Being able to talk directly to the EMS supplier created a feeling of being more involved and having an impact on the development of the system. Some participants spoke with pride about being selected to try out the system and about their contribution to the development of new technology for assessing hand hygiene.

#### Feeling supportive leadership

Co-workers experienced support and encouragement from leaders on the ward. Through their engagement and commitment, the leaders were inspiring and created a positive feeling about the project (Table 2: Q9, Q10).

Regular information about the EMS and the progress of the project was well received, even though a few co-workers expressed a need for even more information. However, some co-workers experienced a lack of encouragement from the leaders during a couple of months before the interviews were conducted (Table 2: Q11). This was explained by an extraordinarily demanding and stressful time on the ward due to an increasing number of patients and several new employees.

### Knowing and confirming

During the analysis, two sub-categories from the perspectives of all HCWs (leaders and co-workers) involved in the implementation of the EMS emerged.

• Acknowledging the importance of hand hygiene
• Being aware of the relationship between hand hygiene and patient safety

#### Acknowledging the importance of hand hygiene

Both leaders and co-workers were aware of the fact that well-performed HH prevents the spread of pathogens and they spoke of HH as an important factor to prevent infections (Table 2: Q12).

It was often mentioned that all the new HCWs on the ward received information about the hospital’s hygiene routines, including HH routines, as an introduction to working on the ward. In addition to this, they also spoke of a short digital course (about 1 hour) provided by the hospital for all HCWs every year, as a way to update their knowledge of hygiene routines. Additional information on hygiene was sought by them, if necessary, within the hospital hygiene routines, or on the internet. Moreover, it was said that the implementation of the EMS had led to many more discussions and everyday talk about HH on the ward, although some of them requested even more opportunities for discussion (Table 2: Q13).

#### Being aware of the relationship between hand hygiene and patient safety

Hygiene routines were discussed as an important safeguard for patient safety, but the concept of patient safety comprised far more than just hygiene routines. For example, HCWs spoke about safe medication administration, safe blood transfusion, preventing the risk of patients falling, preventing pressure ulcers and securing patient confidentiality (Table 2: Q14).

A few HCWs expressed doubts and discussed whether HH alone was enough to prevent HAIs. However, within the implementation project, the HH aspect was seen as the most important.

### Collaborating for progress

The categories *leading and facilitating, participating and contributing* and *knowing and confirming* interrelate and affect each other and, when these categories were merged, the core category emerged as *collaborating for progress*. Leaders are optimistic and committed to the implementation of the EMS, trying to facilitate and support their co-workers in the process of implementation. At the same time, the co-workers are positive about the support they receive and contribute by raising questions and demands for the product to be used in clinical settings. In addition, the involved HCWs understand the objective of the implementation. Together, these aspects indicate forward-thinking to enable the implementation, defined as *collaborating for progress*.

## Discussion

The present study provides insights into the way HCWs experience the implementation of new HH technology, filling an important gap in knowledge that is useful for future infection-prevention technology implementation.

Implementing technical innovations in healthcare is complex and demanding (Conway, 2016; Phillips, 2019; Ward et al., 2014). Two research approaches have been identified when exploring the implementation and use of technology in healthcare: *technology adoption theories* and *implementation science* (Schoville and Titler, 2015). Technology adoption theories focus on how the end users adopt technology and come to accept and use the technology in their daily work (Davis, 1989; Schoville and Titler, 2015), while theories within implementation sciences focus on understanding the complex context in which the innovation is going to be implemented, as well as defining which implementation strategy will work and why (Nilsen, 2015). Technology adoption has been investigated in several previous studies on EMSs (Dyson and Madeo, 2017; Granqvist et al., 2021; Tarantini et al., 2019). However, few studies have applied implementation theories to interpret or guide the implementation of an EMS (Boscart et al., 2012). In the present study, the social and organizational context is central within the findings. The leaders and co-workers collaborated jointly for progress to enable the implementation of the EMS, revealing leadership, participation and knowledge as central aspects.

Social and organizational contexts are examined and analyzed in several implementation theories, one of which is the Normalization Process Theory (NPT) (May and Finch, 2009). Looking at the findings from the perspective of NPT can help us to further understand the result. The NPT comprises four elements that are necessary for successful implementation: coherence (sense-making), cognitive participation (engagement), collective action (work undertaken to implement the intervention) and reflexive monitoring (appraisal work of the advantages and disadvantages of the intervention) (May and Finch, 2009). The present study implies that the participants were aware of the objective of the implementation (coherence). Understanding the importance of HH and the objective of the HH innovation has been described previously as an important factor for the successful implementation of an EMS (Kelly et al., 2021).

We identified leaders on the ward as important key participants, who created a community around the EMS by supporting co-workers and being present on the ward to answer queries and questions (cognitive participation). This reiterates the findings from several previous studies, along with WHO recommendations; the successful implementation of HH innovations requires leadership and especially leadership from all levels of the organization (Drey et al., 2020; Pittet et al., 2000; World Health Organization, 2009).

The demands and requests from the co-workers were taken seriously by the ward leaders and they were collectively able to ensure that the implementation process continued (collective action). One inhibiting factor, an increased workload, was mentioned. It affected the focus on the implementation of the EMS. Clinical work pressure has repeatedly been shown to hinder adherence to HH among healthcare workers (Chang et al., 2022; Scheithauer et al., 2017; Smiddy et al., 2015). Our findings imply that an increased workload affected the engagement and therefore interfered with the implementation process of HH technology innovations.

Feedback at group level from the EMS was used by the leaders to highlight HH adherence on the ward, as a way of promoting the effects of the EMS (reflexive monitoring). Prior studies have noted that feedback from HH technology increases awareness of HH, but they also point out that feedback data need to be credible and reliable (Granqvist et al., 2021; Kelly et al., 2021).

Context and contextual differences can affect how successful and effective HH interventions are and this has been discussed in several previous studies (Conway, 2016; Drey et al., 2020; Gould et al., 2017). Ward culture, organizational and social context influence the success of interventions designed to improve HH adherence (Conway, 2016; Drey et al., 2020). The NPT (May and Finch, 2009) helps to identify the dynamic collective work and the relationships involved during the implementation of the EMS, in the specific context described in the present study.

### Limitations

The present study was conducted on a single surgical ward and only RNs and NAs who had volunteered to be users of the EMS were interviewed. This could possibly have affected the outcome in a more positive direction, as participants might have been in favour of the EMS from the beginning. Future HH technology implementations should aim to involve all HCWs involved in patient care, as they are all obliged to follow HH routines and are therefore potential end-users of the technology.

One advantage of the present study is that it included individual, pair and group interviews, according to the participants’ own choice. Being able to choose how to be interviewed can help participants to talk more freely during interviews, which is preferable in the GT method (Strauss and Corbin, 2015).

Qualitative research does not strive for objectivity in the same way quantitative research does. The researchers’ own interpretations of the collected data will always be present and, for this reason, being impartial and treating data with sensitivity is crucial. GT by Strauss and Corbin (2015), with its rigorous approach to data analysis, has strategies to deal with bias. Through a systematic research process, interpretations are minimized, thereby generating a trustworthy result.

GT aims to generate a theory through data analysis (Strauss and Corbin, 2015). The present study identified a core category explaining how the HCWs collaborated for progress when implementing an EMS in a clinical context. A core category can be used in future studies to produce a general theory of the explored phenomenon (Strauss and Corbin, 2015).

## Conclusion and implications for future research

The present study revealed that HCWs collaborated for progress, that is, they were all included and engaged in the progression of the implementation of the EMS. In the context of NPT, the findings imply that several collective actions were taken by the HCWs to ensure the implementation in the clinical setting. Leadership, participation and knowledge were identified as central and crucial aspects.

In the future, when implementing new technology, such as an EMS, we strongly recommend involving both leaders and users of the technology to promote a successful implementation in order to enhance the collaborative process.

## Supplemental Material

Supplemental Material - Central aspects when implementing an electronic monitoring system for assessing hand hygiene in clinical settings: A grounded theory study Supplemental Material for Central aspects when implementing an electronic monitoring system for assessing hand hygiene in clinical settings: A grounded theory study by Karin Granqvist, Linda Ahlstrom, Jon Karlsson, Birgitta Lytsy and Annette Erichsen in Journal of Infection Prevention

## Data Availability

Data analysed during the current study are available from the corresponding author in response to a reasonable request.
