# Supplementary material for: Central aspects when implementing an electronic monitoring system for assessing hand hygiene in clinical settings: A grounded theory study
Source: J Infect Prev. 2024 Feb 5;25(3):51–8. doi: 10.1177/17571774241230678 (PMC10998548; doi:10.1177/17571774241230678)
Supplement: Supplemental Material - Central aspects when implementing an electronic monitoring system for assessing hand hygiene in clinical settings: A grounded theory study [file sj-pdf-1-bji-10.1177_17571774241230678.pdf]

SUPPLEMENTARY FILE

Central aspects when implementing an electronic monitoring system for assessing hand hygiene in clinical settings: A grounded theory study

Data analysis step by step

An interview guide was developed within the research team (KG, AEA, LA) to address different topics during the interviews relating to HCWs' experiences of the implementation of the EMS on the ward. The interviews aimed to have the characteristics of a dialogue to encourage participants to talk freely and the interview guide helped the interviewer to address different topics throughout the session. All the interviews took place in connection with a working shift but in a quiet, undisturbed room.

As in the basic description of grounded theory research (Strauss and Corbin, 2015), memos were written by the interviewer during each interview and consisted of a summary of the discussions between participants and interviewer before and after the interviews, as well as immediate impressions and thoughts from each interview moment. These memos were then discussed within the research team and included in the analysis to enable theoretical sampling in order to obtain a further understanding of the explored phenomenon.

The data consisted of audio-recorded interviews, transcripts and memo notes. The first interview was conducted as a pilot interview with one participant, evaluated within the research team (KG, LA, AEA) and subsequently included in the analysis. Five interviews were then conducted and analyzed one after the other. The preliminary results of these interviews were then discussed and reflected upon before the remaining eight interviews were carried out.

The interviews were listened to and read through repeated times by the research team (KG, AEA, LA). As in the GT method, the analysis was performed as an iterative process via

constant comparison of the data and the analysis was carried out in three steps: initial coding, axial coding and selective coding. First, initial coding helped to identify codes from each sentence/line of the textual data and to group similar codes to form lower-level categories. During the axial coding, relationships between lower-level categories were examined to produce more refined categories. By merging these refined categories throughout the selective coding, the core category emerged and could be defined. Strauss and Corbin's paradigm model and conditional matrix (Strauss and Corbin, 2015) helped to explore the characteristics and the relationship(s) between categories during the analytic process. Memos were constantly discussed and reflected upon during the analysis to obtain a further understanding of the findings. Constant comparisons of data in all the steps of the analysis process helped the researchers (KG, LA, AEA) to define when categories were saturated. To ensure consensus during the analysis process, any discrepancies while examining and coding data were discussed and reflected upon. Finally, additional researchers (JK, BL) were involved in finalizing the analysis to ensure consistency in the application of data.

Reference

Strauss AL and Corbin JM (2015) *Basics of qualitative research : techniques and procedures for developing grounded theory.* Los Angeles : SAGE.

Standards for Reporting Qualitative Research (SRQR)*

http://www.equator-network.org/reporting-guidelines/srqr/

Title and abstract

	Reported section
**Title** - Concise description of the nature and topic of the study Identifying the study as qualitative or indicating the approach (e.g., ethnography, grounded theory) or data collection methods (e.g., interview, focus group) is recommended	**Title**
**Abstract** - Summary of key elements of the study using the abstract format of the intended publication; typically includes background, purpose, methods, results, and conclusions	**Abstract**

Introduction

**Problem formulation** - Description and significance of the problem/phenomenon studied; review of relevant theory and empirical work; problem statement	**Background**
**Purpose or research questio**n - Purpose of the study and specific objectives or questions	**Background**

Methods

**Qualitative approach and research paradigm** - Qualitative approach (e.g., ethnography, grounded theory, case study, phenomenology, narrative research) and guiding theory if appropriate; identifying the research paradigm (e.g., postpositivist, constructivist/ interpretivist) is also recommended; rationale**	**Method:** Design
**Researcher characteristics and reflexivity** - Researchers' characteristics that may influence the research, including personal attributes, qualifications/experience, relationship with participants, assumptions, and/or presuppositions; potential or actual interaction between researchers' characteristics and the research questions, approach, methods, results, and/or transferability	**Authors**<br>**Method:** Design<br>**Method:** Data collection<br>**Method:** Data analysis
**Context** - Setting/site and salient contextual factors; rationale**	**Method:** Context and implementation of the electronic monitoring system<br>**Method:** The innovation – the electronic monitoring system<br>**Method:** Participants

**Sampling strategy** - How and why research participants, documents, or events were selected; criteria for deciding when no further sampling was necessary (e.g., sampling saturation); rationale**	**Method:** Data collection
**Ethical issues pertaining to human subjects** - Documentation of approval by an appropriate ethics review board and participant consent, or explanation for lack thereof; other confidentiality and data security issues	**Declarations:** Ethical approval and consent to participate<br>**Declarations:** Consent for publication
**Data collection methods** - Types of data collected; details of data collection procedures including (as appropriate) start and stop dates of data collection and analysis, iterative process, triangulation of sources/methods, and modification of procedures in response to evolving study findings; rationale**	**Method:** Data collection<br>**Method:** Data analysis
**Data collection instruments and technologies** - Description of instruments (e.g., interview guides, questionnaires) and devices (e.g., audio recorders) used for data collection; if/how the instrument(s) changed over the course of the study	**Method:** Data collection<br>**Method:** Data analysis
**Units of study** - Number and relevant characteristics of participants, documents, or events included in the study; level of participation (could be reported in results)	**Method:** Participants<br>**Method:** Data collection
**Data processing** - Methods for processing data prior to and during analysis, including transcription, data entry, data management and security, verification of data integrity, data coding, and anonymization/de-identification of excerpts	**Method:** Data collection<br>**Method:** Data analysis
**Data analysis** - Process by which inferences, themes, etc., were identified and developed, including the researchers involved in data analysis; usually references a specific paradigm or approach; rationale**	**Method:** Data analysis
**Techniques to enhance trustworthiness** - Techniques to enhance trustworthiness and credibility of data analysis (e.g., member checking, audit trail, triangulation); rationale**	**Method:** Data analysis<br>**Method:** Data collection

Results/findings

**Synthesis and interpretation** - Main findings (e.g., interpretations, inferences, and themes); might include development of a theory or model, or integration with prior research or theory	**Results**
**Links to empirical data** - Evidence (e.g., quotes, field notes, text excerpts, photographs) to substantiate analytic findings	**Results**

Discussion

**Integration with prior work, implications, transferability, and contribution(s) to the field -** Short summary of main findings; explanation of how findings and conclusions connect to, support, elaborate on, or challenge conclusions of earlier scholarship; discussion of scope of application/generalizability; identification of unique contribution(s) to scholarship in a discipline or field	**Discussion**
**Limitations** - Trustworthiness and limitations of findings	**Discussion**:

	Limitations

Other

**Conflicts of interest** - Potential sources of influence or perceived influence on study conduct and conclusions; how these were managed	**Declarations:** Conflict of interest<br>**Declarations:** Funding
**Funding** - Sources of funding and other support; role of funders in data collection, interpretation, and reporting	**Declarations:** Funding

*The authors created the SRQR by searching the literature to identify guidelines, reporting standards, and critical appraisal criteria for qualitative research; reviewing the reference lists of retrieved sources; and contacting experts to gain feedback. The SRQR aims to improve the transparency of all aspects of qualitative research by providing clear standards for reporting qualitative research.

**The rationale should briefly discuss the justification for choosing that theory, approach, method, or technique rather than other options available, the assumptions and limitations implicit in those choices, and how those choices influence study conclusions and transferability. As appropriate, the rationale for several items might be discussed together.

Reference:

O'Brien BC, Harris IB, Beckman TJ, Reed DA, Cook DA. **Standards for reporting qualitative research: a synthesis of recommendations.** *Academic Medicine*, Vol. 89, No. 9 / Sept 2014
DOI: 10.1097/ACM.0000000000000388
